# A filling rig for liquid and gas working fluids for two-phase thermal management systems

**DOI:** 10.1016/j.ohx.2024.e00558

**Published:** 2024-07-11

**Authors:** Colin Butler, Emmanuel Caplanne, Jeff Punch

**Affiliations:** a Stokes Laboratories, Bernal Institute, School of Engineering, University of Limerick, Limerick, Ireland; b Thermal Control Section (TEC-MTT), European Space Agency (ESA), ESTEC, Noordwijk, Netherlands

**Keywords:** Heat pipes, Thermal control, Two-phase cooling, Ammonia, Vapour transfer

## Abstract

Two-phase cooling devices are used to remove and dissipate heat from high power-density electronic systems to maintain them within their operating temperature limits. The manufacture of these devices, such as heat pipes, thermosyphons or vapour chambers, involves firstly removing any internal air or non-condensable gases before charging with the required volume of working fluid. This paper presents detailed designs and operating instructions for a single bench-top station for use in a laboratory environment for the vacuum evacuation, degassing and charging of these devices. Two configurations allow for the filling of fluids which are either liquids or gases at standard temperature and pressure conditions. For liquids, the dispensed volume can be measured directly on an integrated burette, while the method of vapour transfer is used for gases.

The hardware was demonstrated by filling multiple thermosyphon devices with a number of common working fluids used in two-phase systems, including water, acetone and ammonia. It was shown to deliver precise and repeatable filling volumes with average differences compared to target volumes of 1.7% and 10.5% for liquids and gases respectively. The design is intended to be highly customisable where its size can be modified to accommodate filling volume requirements for different applications.

## Graphical abstract

**Figure:**
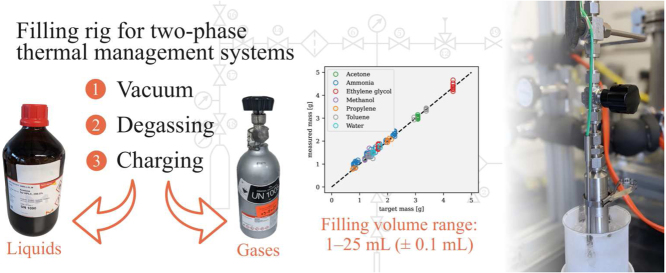

## Specifications table

**Table:** 

Hardware name	Two-phase filling rig
Subject area	Engineering and material science
Hardware type	Mechanical engineering and materials science
Closest commercial analog	No commercial analog is available
Open source license	CC BY-SA 4.0
Cost of hardware	€5,000–€15,000 (depending on configuration and vacuum pump used)
Source file repository	https://doi.org/10.17632/bjkbjtf3vk

## 1 Hardware in context

The use of two-phase heat transfer systems for cooling is a well established technology in the field of thermal management and control. Processes which make use of boiling and condensation of a working fluid through the inclusion of latent heat can achieve significantly larger rates of heat transfer with smaller temperature differences when compared to other processes without phase change [1]. Examples of two-phase devices include heat pipes, loop heat pipes and heat pumps. New designs and configurations are continuously being developed to improve the efficiency and sustainability of high energy-density and power-density devices for a wide range of engineering applications such as consumer electronics, data centres, spacecraft, and nuclear reactors [2], [3], [4], [5], [6].

In order to manufacture any two-phase cooling device, the internal air, as well as any non-condensable gases dissolved inside the intended working fluid, must be firstly removed to ensure correct operation. Failure to do so can cause sub-optimal thermodynamic conditions (such as the internal pressure), corrosion of the metal container, or lead to gas blockages in the condenser region, thereby limiting the rate of heat transfer or causing overall device failure [7]. This can significantly lower the lifetime of any system which the heat transfer device is being used to cool. The simplest approach for filling the working fluid into a device, such as a copper–water heat pipe, would be to pour in the required volume of liquid and then raise its temperature using an external heater until the liquid starts to boil. The resulting vapour forces any air out of the pipe and it is then quickly sealed before too much of the working fluid is lost. In refrigeration or HVAC systems, which may use high-pressure synthetic refrigerants, a recovery pumping unit is used to transfer the working fluid between a supply tank and the heat transfer device. Both of these methods have the distinct disadvantage of lack of control over the precision and accuracy of the dispensed volume, as well as not performing any purification or degassing of the working fluid before filling. The working fluid filling ratio, i.e., the ratio of the liquid volume to the volume of the evaporator section, plays a pivotal role in two-phase systems to ensure that they operate safely and efficiently [8]. The preferred approach generally adopted in literature is the use of a rig which may be used to perform working fluid degassing and metering, as well as degassing of the heat transfer device [9], [10]. However, filling rig designs for working fluids which can be either liquids or gases at ambient conditions are generally not provided and detailed descriptions are notably absent in current literature. Studies related to experimental testing of two-phase devices instead tend to focus on their design improvements and the resulting heat transfer performance. The aim therefore of this paper is to provide detailed designs and methodologies for a filling rig which uses either liquids or gases as the working fluid which is intended for bench-top use in a laboratory environment.

## 2 Hardware description

**Fig. 1:**
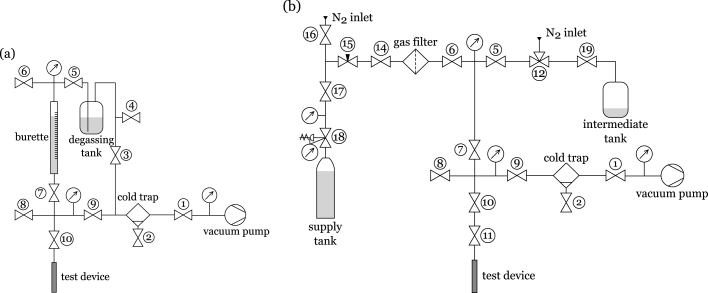
Filling rig schematic diagrams (a) liquid configuration (b) gas configuration.

The filling rig detailed in this paper was designed to act as a single station to allow for both the degassing and charging of two-phase heat transfer devices with their required working fluid volume. Fig. 1 presents the schematics for the design. It incorporates some features from previously published rigs in literature [9], [11] but most significantly allows for the filling of fluids which are either liquids or gases at standard temperature and pressure (STP) conditions. This was achieved by having a section which is common for both fluid states, but then two separate configurations for dispensing the fluid inside the heat transfer device. The common section includes the vacuum pump and related hardware, as well as the attachment point for the heat transfer device to be filled (i.e. the bottom section in the diagrams in Fig. 1 from the vacuum pump up to valve ⑦). For the two configurations, liquid or gas, a choice can be made to build either top section depending on the final application, or indeed, both configurations can be employed whereby a changeover between them is straightforward to achieve with the current design. After any changeover between configurations (or working fluids), leak testing and bake-out of the full system should be performed to reduce contamination and improve the ultimate vacuum level.

For the liquids, a sealed glass flask is used to visualise the degassing step before it is transferred into a graduated glass burette for dispensing. The method used to reduce the concentration of dissolved gases from the liquids involves repeatedly pulling a vacuum on the vapour/air above the liquid [12]. Using this technique with acetone as an example, a vacuum needs to applied at least four times to the glass flask to lower the concentration of O_2_, N_2_ and CO_2_ to less than 3 ppm. The advantage of this method is that because the internal pressure is never below the vapour pressure of the liquid, high performance vacuum pumps are not needed to thoroughly degas the liquid.

In the case of gases, the method of vapour transfer or distillation is used to transfer the fluid from a supply tank. A purifier is used for maintaining high purity levels of the gas working fluid. Anhydrous grades of gases have a purity level of ≥99.98%, with impurities typically consisting of ≤200 ppm of moisture and ≤5 ppm of oil [13]. These contaminates, if not adequately removed before filling, can lead to significant non-condensable gas blockages inside the two-phase device, and the presence of water in the supply can corrode aluminium or stainless steel materials [10]. The vapour transfer method prevents the transfer of oils, while the gas purifier has the advantage of removing the impurities directly from the gas stream. Other solutions in place of the purifier would be the use of ultra high purity grade (≥99.99999%) which can cost an order of magnitude more than the anhydrous grade, or a freeze-pump-thaw method could be implemented which works in a similar fashion to liquid degassing but requires the use of liquid nitrogen to freeze the fluid before vacuum is applied.

**Fig. 2:**
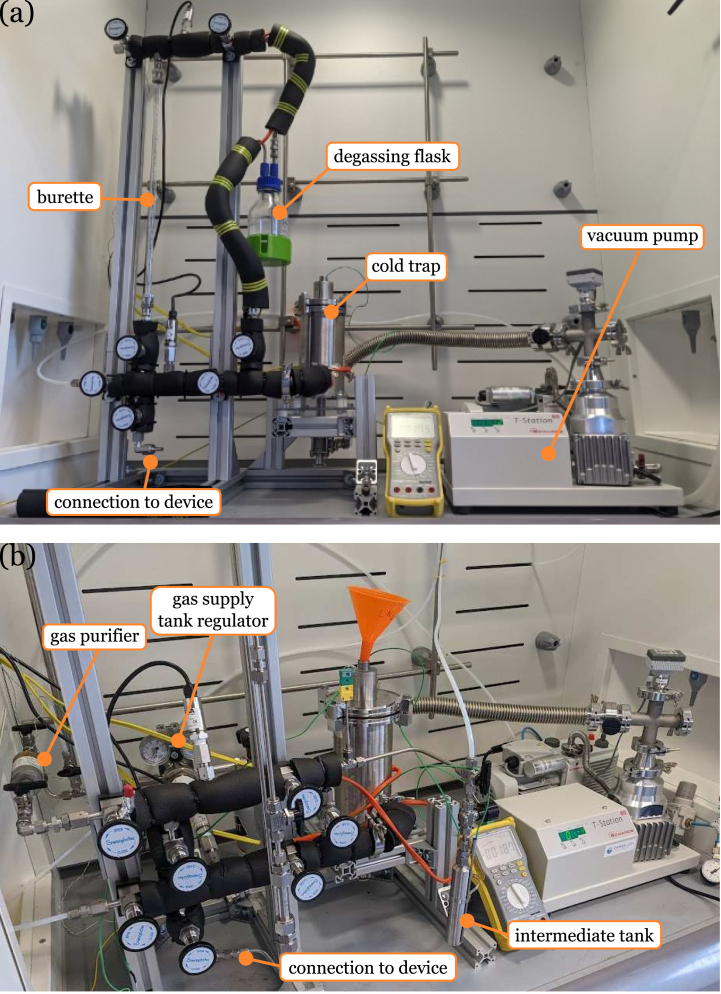
Filling rig inside laboratory fume cupboard (a) liquid configuration (b) gas configuration.

The hardware described was designed for the intended application of the precise and repeatable filling of small-to-medium sized heat transfer devices, such as thermosyphons, heat pipes or vapour chambers, with liquid filling volumes in the range of 1–25 mL. However, this design can be completely customised by substituting some of the different components to match filling volume requirements. As well this, the overall rig was designed to be bench-top scale and fit within the confines of a standard laboratory fume cupboard for cases where the intended working fluid may be hazardous for laboratory users, such as toluene or ammonia. Fig. 2 shows the two different configurations set up inside a fume cupboard with some of the main elements highlighted. The size and customisability of this design allows for the rapid development and prototyping of two-phase thermal management devices.

In summary, the test rig design presented here is:

• A single bench top scale station for the combined degassing, purification and dispensing of working fluids (either liquids, gases or both) for the filling of two-phase heat transfer devices.
• Fully customisable and scalable whereby the design can be modified to allow for different working fluid filling volumes or operating pressures outside of those described.
• Applicable to other laboratory tasks, such as the manipulation of air or moisture sensitive samples, similar to a Schlenk line.

## 3 Design files summary

The files associated with the design are listed in Table 1.

Below is a brief description of the design files:

**Table 1:** Design files.

Design filename	File type	Open source license	Location of the file
Assembly drawings.pdf	CAD file	CC BY-SA 4.0	https://doi.org/10.17632/bjkbjtf3vk
flaskHolder.stl	3D printing	CC BY-SA 4.0	https://doi.org/10.17632/bjkbjtf3vk
valveFixture.FCStd	CAD file	CC BY-SA 4.0	https://doi.org/10.17632/bjkbjtf3vk
thermalSpacer.FCStd	CAD file	CC BY-SA 4.0	https://doi.org/10.17632/bjkbjtf3vk
Electrical schematics.pdf	CAD file	CC BY-SA 4.0	https://doi.org/10.17632/bjkbjtf3vk
pressureRead.py	Software	CC BY-SA 4.0	https://doi.org/10.17632/bjkbjtf3vk

• *Assembly drawings.pdf* contains CAD models of the two different configurations, along with the associated schematic diagrams and part designators corresponding to the bill of materials table, also provided in this file. Note that the CAD files for each of the individual pressure or vacuum fittings are available in different formats directly from the vendors’ website.
• *flaskHolder.stl* is a file for the 3D printing of the component used to mount the glass flask to the aluminium profile frame. It was designed to fit a 500 ml Duran style laboratory bottle and was 3D printed with PLA filament.
• *valveFixture.FCStd* is the 3D CAD file of the components used to mount the Swagelok bellow sealed valves to the aluminium profile frame. The countersunk holes in each part correspond to the mounting hole pattern provided in the valves’ datasheet, and the slotted hole is used for alignment along the aluminium profile. It was machined from PTFE to offer good heat resistance.
• *thermalSpacer.FCStd* is the 3D CAD file of the components used to mount the cold trap to the aluminium profile frame and was used in order to minimise heat conduction between the two. It was machined from PTFE to due its low thermal conductivity.
• *Electrical schematic.pdf* contains the electrical connection diagrams and bill of materials tables for bake-out heater and controller, and for reading pressure data from the transducers.
• *pressureRead.py* is the Python script used to read and record the data from the pressure transducers.

## 4 Bill of materials summary

The bill of materials is provided in the assembly drawing files described above. Each component has a bill of materials number and valves are also given a number corresponding to the diagrams in Fig. 1 which are referred to in Section 6 to describe the system operation. The quantities are listed per configuration so only the materials for the selected fluid type are required. As this design can be customised to a particular application or filling volume, the parts list does not need to be strictly adhered to. As examples, a burette with a different total volume or increment sizes can be used instead; the pressure transducers can be substituted with different models or with pressure gauges depending on the expected operating pressures (the models listed in the bill of materials were used with ammonia as the working fluid which has a vapour pressure of approximately 8.6 bar at a temperature of 20 °C); or a different model of vacuum pump could be used. Additionally for the electronics, if no bake-out is considered necessary, and pressure gauges are used instead of transducers, none of the materials listed are required.

Other materials such as chemicals and working fluids are excluded from the bill of materials as the quantities will depend on the application and number of devices to be filled.

## 5 Build instructions

**Fig. 3:**
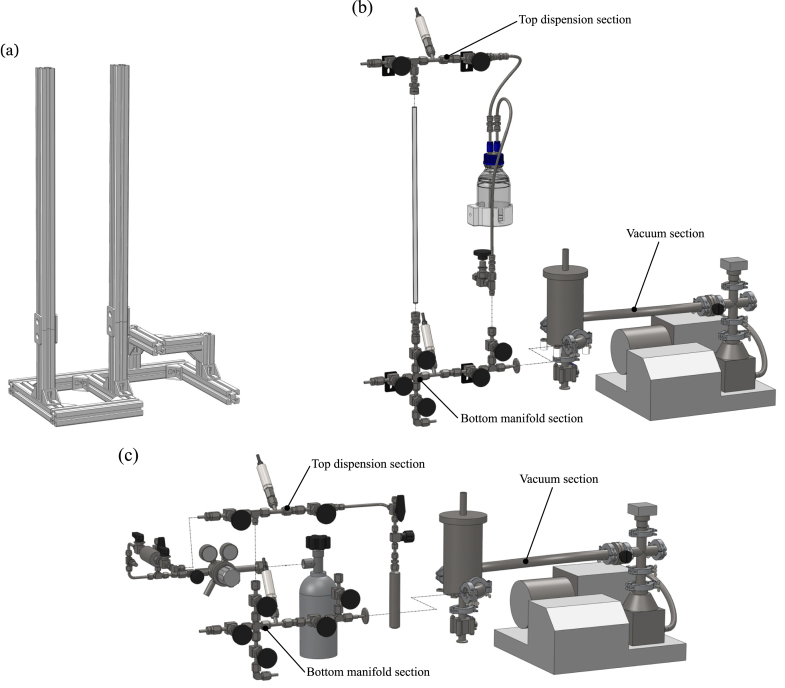
Filling rig assembly (a) aluminium frame (b) exploded view of liquid configuration sections (c) exploded view of gas configuration sections.

As it makes use of vacuum, the fittings and pipework used for construction were either stainless steel or glass. For stainless steel, either ISO-KF fitting were used for the vacuum pump side, or Swagelok VCR metal gasket seal and Swagelok Ultra-Torr O-ring fittings were used in the filling side. The VCR fittings are rated for leak-tight sealing from vacuum (4 × 10^−9^ mbar L/s) to positive pressure (>200 bar depending on fitting type and size) and Ultra-Torr fittings provide vacuum sealing only (4 × 10^−9^ mbar L/s). For the Swagelok fittings, 14 inch size tubing and fittings was selected as a trade-off between pump-down speed and to minimise the amount of liquid wasted between the filling of each device (this process is described later in Section 6). The stainless steel fittings were assembled following the manufacturer guidelines in individual sections before being mounted to the aluminium profile frame. After final assembly, the system should be tested to minimise any leaks. The different sections are described below and shown in Fig. 3.

### 5.1 Aluminium profile frame

The aluminium frame consists of 40 × 40 mm cross section extruded profile and is shown in Fig. 3(a). It was assembled using associated right-angle brackets, M8 bolts and T-nuts. The same frame is used for both liquid and gas configurations. The two largest vertical extrusions are used to support the Swagelok fittings and can be disassembled if required to aid with transferring the rig into the fume cupboard after final assembly is complete.

### 5.2 Vacuum section

The vacuum section consists of the pumping station, cold trap and all associated KF fittings, as shown in Fig. 3(b). The vacuum pump listed in the bill of materials is an Edwards Vacuum 85H T-station and includes both a roughing and turbo-molecular pump with a specified ultimate vacuum level of <5 × 10^−9^ mbar. It also includes a controller used in combination with the vacuum convection gauge (pressure measurement range of 3 × 10^−4^–1333 mbar). This compact system was chosen for its vacuum pumping characteristics to ensure maximal outgassing and removal of contaminates when changing to a different device or working fluid. As the method of vacuum degassing is used to remove dissolved gases from the liquid, any pump which operates below the expected vapour pressure of the working fluid could however be used in its place.

A liquid nitrogen (LN2) cold trap was placed between the pump and the rest of the system in order to prevent any working fluid vapours flowing back and potentially damaging the pump. The cold trap was mounted to the aluminium profile frame using the PTFE machined thermal spacer parts and nylon bolts with the aim of minimising conductive heat transfer to the frame. Valve ② was used to drain any condensate inside the trap after all filling was complete.

### 5.3 Bottom manifold section

The bottom section which can be seen in Fig. 3(b) and (c) acts as a manifold between the vacuum section, working fluid dispension top section and heat transfer device to be filled. It consists of VCR fittings and it is important that they are assembled at right angles relative to each other and aligned with the aluminium profile frame. This section was connected to the frame using the PTFE machined valve fixture parts which include holes corresponding to the mounting hole pattern on the base of the valves. It was free to slide along the aluminium profile and, after alignment of the VCR-to-KF adapter to the vacuum section, it was fixed in place by tightening the corresponding bolts and T-nuts of the valve fixture part.

### 5.4 Top dispension section

The top section is used for working fluid purification and dispension but utilises different components depending on the configuration.

For liquids as shown in Fig. 3(b), the glass burette was firstly assembled to the bottom section using the Ultra-Torr O-ring reducing union fitting, and then the pre-assembled top section was carefully slid down into place over the burette. After ensuring the burette was vertical and not under any bending stresses, the top section was fixed in place by tightening the corresponding bolts on the PTFE valve fixture parts. If bending is observed, the stainless steel parts should be adjusted to minimise the risk of breaking this glass part. Afterwards, the Ultra-Torr fittings were tightened to create the seal around the external surface of the burette. If the standard fluorocarbon (FKM) O-rings are incompatible with the liquid in use (such as acetone or toluene), they should be replaced with a more chemically resistant material, such as perfluoroelastomer (FFKM). The 3d printed flask holder part was assembled to the frame using an M8 bolt and T-nut, and this was used to hold the 500 mL Duran Flask in place. Pressure plus vacuum resistant flasks were used for additional safety. A cap with two ports was used for two pieces of 14 inch stainless steel tubing. As shown in the schematic in 1(a), the longer tube extends to the bottom of the flask/degassing tank where its opening should be fully submerged in liquid, whereas the shorter tube should always stay in the air/vapour space above the liquid. The shorter tube was connected via convoluted flexible metal tubing to the vacuum pump at valve ③ and the longer tube was connected via convoluted flexible metal tubing to valve ⑤.

To changeover between the liquid and gas configuration, all glassware and low pressure O-ring fittings were removed, and only fittings with high pressure ratings were used. The maximum operating pressure expected during filling of gases will be their corresponding vapour pressure at ambient temperature so all fittings and components such as transducers should be selected to ensure they meet an adequate minimum pressure safety rating. This top section for gases can be seen in Fig. 3(c). It was lowered along the aluminium profile frame and connected directly to the bottom section at valve ⑦. Gas was supplied from lecture bottle cylinders (approximate fluid mass of 300 g) but any size gas cylinder can be used. The supply tank was connected to the system via a gas regulator. An Entegris gas purifier was installed to improve final gas purity levels and remove potential contaminants in the supply tank to below part-per-billion (ppb) levels. In order to set the specified maximum flow rate through the purifier, a metering valve ⑮ was placed between it and the supply tank to act as a flow restrictor. The ball valve ⑭ was required for the purifier conditioning procedure. The intermediate tank consists of a stainless steel sampling cylinder with an internal volume of 50 mL. This tank was used during the filling process to condense a fixed volume of working fluid so enough space should be retained around the outside of this tank to allow for it to be submerged in a cooling bath. For purging purposes, inert nitrogen gas can be introduced into the test rig at two locations at valves ⑫ and ⑯.

### 5.5 Electronics

As stated earlier, the electronics are only required if pressure transducers are used for reading and recording pressure data, or for bake-out heaters. The test rig included two Omega PX309-300A5V pressure transducers (0 to 21 bar(a) range, 0 to 5 Vdc output) and they were wired as shown in the electrical schematic diagrams. A Raspberry Pi 3B+ was used to run the included Python script to read and record the data from the transducers. Trace heating tape was used for system bake-out to improve outgassing and decontamination when changing to a new working fluid. The trace heaters were wrapped around the stainless steel sections and secured in place using aluminium conductive tape. A K-type thermocouple was used for temperature feedback to the PID controller. The heating tape was wrapped in polyethylene foam pipe insulation. As this system uses 230 VAC mains power, please be aware of the associated risks and how to minimise them, as well as complying with all relevant safety regulations.

## 6 Operation instructions

As this equipment has two configurations for use with either liquids or gases, they each require a particular set of operating instructions and are detailed below. The circled numbers refer to the valve numbers in Fig. 1 and the assembly drawings file.

### 6.1 Liquid configuration

The following operating procedure was used for the degassing, vacuum evacuation, and charging of a heat transfer device with a liquid working fluid:

1. Open all valves except ②, ④, ⑥, ⑧ and ⑩. Bake-out the system using the vacuum pump and external heating tapes to remove a maximum of residual fluid and contaminates from the system. Any vapours in the system are condensed by the cold trap to prevent them from entering and damaging the pump. The cold trap should be kept topped up with LN2 throughout the filling procedure. Wear appropriate PPE and pour LN2 slowly into the cold trap to prevent it from rapidly boiling. As stated earlier, leaks into the system should be minimised after equipment assembly to prevent the condensation of liquid oxygen inside the cold trap which is a common safety issue associated with Schlenk lines. Close all valves.
2. Half fill the Duran flask/degassing tank with the liquid working fluid (approximately 250 mL). Open valves ① and ③ and remove the air and vapour above the liquid using the vacuum pump. Close valve ③ when boiling of the liquid is observed. No heating of the degassing tank is required during this step [12].
3. Repeat the previous step for the determined number of times to lower the concentration of dissolved gas to the selected amount. See Appendix for more details.
4. Open valve ⑤ to slowly draw the working fluid from the degassing tank into the system and fill the burette using the developed internal pressure differences. If necessary, carefully open valve ④ to use atmospheric pressure to drive the fluid from into the burette. Close valve ④ and open valve ⑥.
5. Attach the test device to the system at valve ⑩. Open valves ⑨ and ⑩. Evacuate and bake-out the test device above its expecting operating temperature. Close valves ⑨ and ⑩.
6. Open valve ⑦.
7. Open valve ⑧ to allow some fluid to drain from the system to ensure the pipework is full of liquid and to decrease the level in the burette such that the graduations are readable. Close valve ⑧.
8. Slowly open valve ⑩ to allow fluid to flow into the test device. Once the desired amount is dispensed based on the burette measurement, close valve ⑩. The burette has graduations of 0.1 mL in this case, which allows for accurate reading of the dispensed volume.
9. Seal the test device and remove it from the filling station.
10. Close valve ⑦.
11. Drain the remaining fluid by opening valves ⑧ and ⑩. Close valves ⑧ and ⑩.
12. Repeat this procedure from step 5 to fill additional test devices with the same working fluid. If the burette is empty, repeat from step 2.

**Fig. 4:**
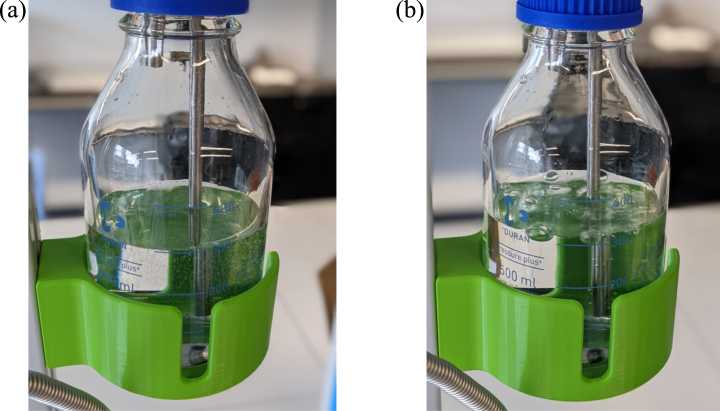
Outgassing of water in Duran flask (a) first vacuum application (b) second or third vacuum application.

During the first degassing of water at step 2, a large quantity of small gas bubbles were observed to be released when vacuum is applied and with repeated applications as the dissolved gas concentration is reduced, working fluid boiling is mainly observed, as shown Fig. 4. See Appendix for more details on how to determine the number of times to apply vacuum for different liquids.

After liquid is transferred into the glass burette and filling of a sample, approximately 7 mL of liquid is wasted after each fill as it must be flushed from the system in order to evacuate the next sample through the vacuum pump. Approximately 2–3 fills can be performed from each full glass burette (total volume of 25 mL) before it must be refilled again from the Duran flask.

### 6.2 Gas configuration

Vapour transfer, or distillation, is used to transfer the fluid from the supply tank to the heat transfer device to be filled. However, in order to measure the amount of working fluid dispensed, a calibration procedure must first be used to determine a relationship between the filling mass and filling time for each working fluid. This is achieved by holding all other parameters in the system constant during this procedure, i.e. inlet gas pressure, internal volume and temperature, and varying the filling time only. The intermediate tank acts as the fixed volume in which the required quantity of working fluid is first condensed. It is then transferred from there into the heat transfer device, which can be of any size. The following procedure is used to perform this calibration and assumes that the gas purifier has been installed and fully conditioned (as per the purifier manual), and there is working fluid gas in the line between the supply tank and valve ⑥:

1. Disconnect the intermediate tank and valve ⑲ from the filling rig between valve ⑫ and ⑲, measure and record their empty mass, and then reconnect them to the rig.
2. Open all valves except ②, ⑥, ⑧ and ⑩. Evacuate the system using the vacuum pump. Close all valves.
3. Chill the intermediate tank in a cooling bath of dry ice and isopropyl alcohol until its temperature reaches steady state at the sublimation temperature of CO_2_ of −78 °C.
4. Set the inlet pressure from the supply tank on the gas regulator (valve ⑱) and open valves ⑰ and ⑭. Note that the metering needle valve ⑮ is opened one turn to set the gas flow rate and left open for the duration of the calibration and filling process. Changing it again will invalidate the calibration as the flow conditions will no longer be identical.
5. Open valves ⑤, ⑥ and ⑫.
6. Open valve ⑲ and immediately start a timer. Ensure the temperature in the cooling bath remains constant and does not increase as condensation takes place inside the intermediate tank. Add more dry ice to the cooling bath if necessary.
7. After 30 s, close valve ⑲ and stop the timer. Remove the intermediate tank from the cooling bath, allowing it to warm to room temperature.
8. Close valve ⑥.
9. Open valves ⑦, ⑧ and ⑫ and purge any remaining working fluid gas from the system using N_2_.
10. Disconnect the intermediate tank and valve ⑲ from the rig between valve ⑫ and ⑲ and record the new mass, then reconnect the intermediate tank and valve ⑲.
11. Purge the working fluid from the tank using N_2_ by opening valve ⑲.
12. Close ⑲.
13. Repeat this procedure from step 2 for different time durations (e.g. 45, 60, 90 s) recording the mass of the intermediate tank and valve ⑲ each time.
14. Generate a curve fit of working fluid mass as a function of time.

**Fig. 5:**
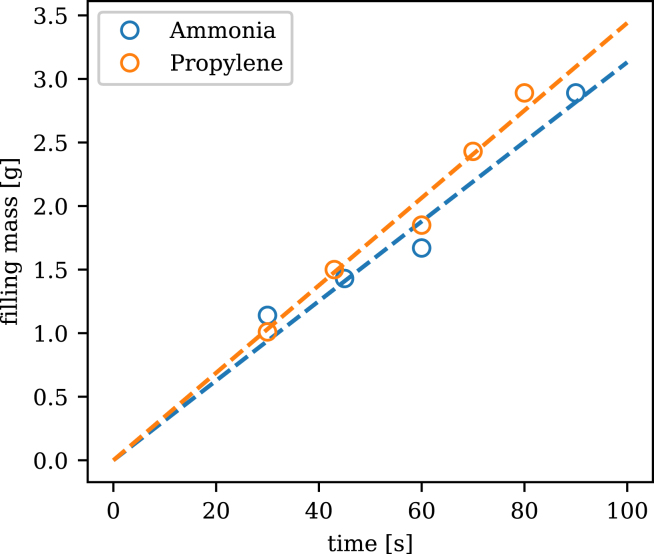
Gas filling calibration curves.

Fig. 5 shows the calibration data measured for ammonia and propylene as working fluids. A least squares linear fit between the filling time and the increase in mass was calculated ($R^{2}$ values of 0.993 and 0.997 for ammonia and propylene respectively) which was then used to determine the corresponding filling time for any mass. The filling volume can be determined from the liquid density at ambient temperature.

The following operating procedure was used for the vacuuming and charging of a test sample with a gas working fluid (Note: this procedure assumes that (a) the gas purifier has been installed and fully conditioned (as per the purifier manual) and there is working fluid gas in the line between the supply tank and valve ⑥, and (b) the calibration has been performed and valve ⑮ has not been adjusted):

1. Attach the test device along with a filling valve ⑪ to the system at valve ⑩.
2. Open valves all valves except ②, ⑥, and ⑧. Evacuate and bake-out the test device above its expecting operating temperature. Close all valves.
3. Chill the intermediate tank in a cooling bath of dry ice and isopropyl alcohol until its temperature reaches steady state at approximately −78 °C.
4. Set the inlet pressure from the supply tank on the gas regulator (valve ⑱) to the same value as that used during calibration, and open valves ⑰ and ⑭.
5. Open valves ⑤, ⑥ and ⑫.
6. Calculate the time duration from the required filling mass using the relationship determined during the calibration.
7. Open valve ⑲ and immediately start a timer. Ensure the temperature in the cooling bath remains constant and does not increase as condensation takes place inside the intermediate tank. Add more dry ice to the cooling bath if necessary.
8. When the time duration from step 6 has been reached, close valve ⑲ and stop the timer.
9. Close valve ⑥, then open valves ⑦, ⑩ and ⑪.
10. Remove the cooling bath from the intermediate tank and use it to now chill the test device.
11. Open valve ⑲. Apply a water heating bath of approximately 35 °C to the intermediate tank to increase the rate of evaporation.
12. Monitor the internal pressure indicated by the transducers. When it stabilises at approximately the working fluid vapour pressure corresponding to the dry ice and isopropyl alcohol cooling bath temperature, close valve ⑪.
13. Open valves ⑧ and ⑫ and purge the system with N_2_. Close valves ⑧ and ⑫.
14. Remove the test device and valve ⑪ from the rig between valves ⑩ and ⑪.
15. Repeat this procedure from step 1 to fill additional test samples with the same working fluid.

## 7 Validation and characterisation

The filling station described in this paper was used during the manufacturing stage of multiple two-phase heat transfer devices using various combinations of both outer metal container and internal working fluid. These included heat pipe and thermospyhon devices with an outer diameter of 12.7 mm and lengths in the range of 180–400 mm. For filling of such devices, the volume of liquid must not be too small a quantity which can lead to evaporator dry-out, or too large a quantity which can completely fill the evaporator and lead to liquid being carried to the condenser, thereby causing blockage of surfaces for phase change [9], [14], [15]. For thermosyphons, the recommended liquid fill should be in the range of 50%–80% of the volume of the evaporator, and also the volume of liquid, $V_{l}$, should be related to its dimensions as follows:

(1) $$V_{l}>0.001\left(d-2t\right)\left(l_{e}+l_{a}+l_{c}\right)$$

where d is the diameter, t is the wall thickness, and $l_{e}$, $l_{a}$ and $l_{c}$ refer to the lengths of the evaporator, adiabatic and condenser sections, respectively. For heat pipes, the general recommendation for liquid volume is that there should be enough to fully saturate the wick plus a small excess [9]. The estimated filling volumes for the different sample lengths used during this study are shown in Table 2.

The filling masses for the different investigated working fluids corresponding to these volumes are shown in Table 3 using density data at 25 °C [16]. In order to verify the final filled volume of a device, its mass (including any attached valve) was measured before and after filling using a Kern EHA 500-2 precision balance. The working fluids listed in Table 3 are among the most commonly used for two-phase devices because of their superior thermodynamic properties (such as density, latent heat of vaporisation, surface tension and viscosity) within temperature ranges applicable to thermal management of electronic devices. Ammonia and proplyene are gases at room temperature while the remain fluids are liquids, so both configurations of the filling rig were utilised.

**Table 2:** Liquid filling volume for sample length.

Sample length [mm]	Fill volume [mL]
180	1.9
200	2.2
310	3.4
400	4.4

**Fig. 6:**
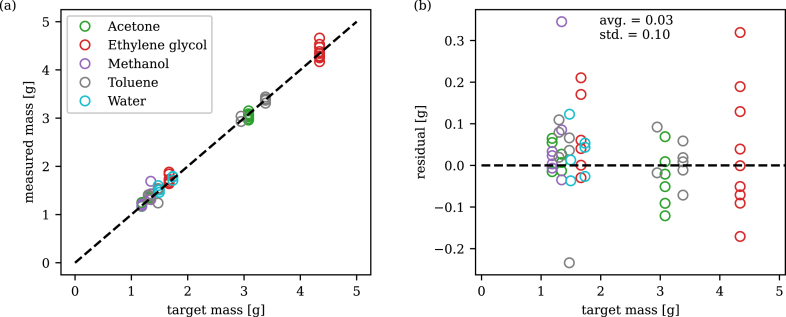
Liquid filling masses (a) measured mass compared to target mass (b) residuals.

Fig. 6 presents the results of the filling process for the different liquids in terms of the target mass versus the measured value after the samples had been evacuated and outgassed using the liquid configuration of the test rig. During operation, the scale on the burette was used to read the filling volume and the final dispensed value was then verified using the measured increase in sample mass. Excellent accuracy and precision was achieved, with an average difference of 0.03 g and a standard deviation of 0.10 g. This corresponds to an average difference and standard deviation of 1.7% and 5.3% compared to the target mass.

**Fig. 7:**
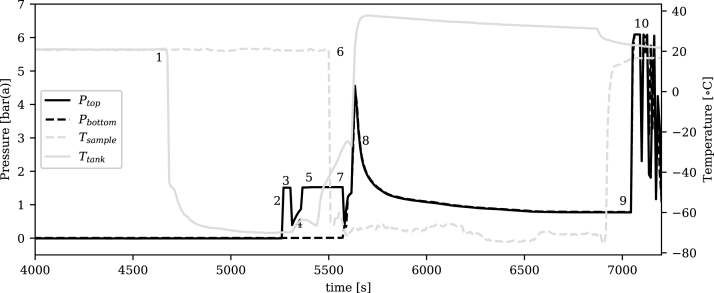
Gas filling process.

**Table 3:** Liquid filling mass at 25 °C.

Fluid	CAS No.	State at STP	Fill mass [g]			
			1.9 mL	2.2 mL	3.4 mL	4.4 mL
Acetone	67-64-1	Liquid	1.49	1.73	2.67	3.45
Ammonia	7664-41-7	Gas	1.15	1.33	2.05	2.65
Ethylene glycol	107-21-1	Liquid	2.11	2.44	3.77	4.88
Methanol	67-56-1	Liquid	1.49	1.73	2.67	3.46
Propylene	115-07-1	Gas	0.96	1.11	1.72	2.23
Toluene	108-88-3	Liquid	1.64	1.90	2.93	3.79
Water	7732-18-5	Liquid	1.89	2.19	3.39	4.39

The rig was then converted to the gas configuration for filling of the ammonia and propylene samples. Fig. 7 illustrates an example of the pressure and temperature measurements recorded during the filling of a sample with propylene. $P_{top}$ and $P_{bottom}$ refer to the pressure transducer readings in the upper and lower sections of the rig respectively, and $T_{sample}$ and $T_{tank}$ refer to the bath temperatures for the sample and the intermediate tank respectively. This plot shows the data at the point in the process after the rig and sample have been evacuated by the vacuum pump for several hours while data acquisition was running, so the x-axis shown does not start at zero.

The process steps labelled in Fig. 7 are described as follows:

1. The intermediate tank was immersed in the dry-ice/isopropyl alcohol cooling bath until its temperature stabilised at approximately −75 °C. The upper and lower sections of the rig were isolated from each other, and valve ⑲ connected to the intermediate tank was closed.
2. Gas was allowed to enter the system at a fixed pressure of 1.5 bar(a) set by the gas supply cylinder regulator. This pressure was the same as that used during the calibration for this fluid.
3. Valve ⑲ at the intermediate tank was opened and a timer was started. The pressure firstly dropped as the gas expanded into the tank.
4. The gas began condensing in the tank and the temperature of the cooling bath started to slowly increase. The pressure also began to slowly increase as new gas enters the rig from the supply cylinder to replace that which was condensing into liquid.
5. After the set time had elapsed, valve ⑲ on the intermediate tank was shut. The gas pressure rose in the remaining areas back to the regulated pressure and the gas inlet at valve ⑥ was shut.
6. The cooling bath was removed from the intermediate tank and its temperature began to slowly increase back towards ambient. The sample was now immersed in the cooling bath and its temperature quickly dropped to <−70 °C. A water bath at approximately 35 °C was placed around the intermediate tank to aid the transfer.
7. Valve ⑦ between the upper and lower sections of the rig was opened connecting the intermediate tank to the sample. After an initial drop in pressure as gas flowed into the evacuated area, it rose rapidly as the water bath caused the liquid to evaporate in the intermediate tank, driving it towards the sample.
8. The pressure dropped as the gas began condensing in the sample. More dry ice was added to the cooling bath to keep its temperature as low as possible.
9. When the pressure stabilised meaning no more gas was condensing, valve ⑪ on the sample was closed. The cooling bath was then removed.
10. Before disconnecting the sample from the filling rig, N_2_ was used to purge any remaining process gas from the system.

**Fig. 8:**
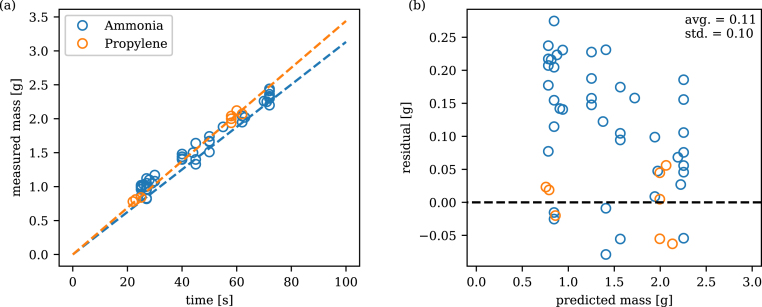
Gas filling masses (a) measured mass as a function of time (b) residuals.

Similar to the liquids, the filling volumes for the gases were verified by measuring the difference in sample mass before and after filling, and after the sample had warmed back to ambient temperature and was dried of any water vapour which had condensed on the external surface from the environment. Fig. 8 shows the final filled volumes compared to the previously determined linear calibration curves for both gases. As can be seen from the plot of residuals, very good agreement was achieved between the target and measured mass. In general, the samples can be seen to be slightly overfilled by an average of 0.11 g (10.5% compared to the target mass) which was attributed to short delays in either manually starting the timer or closing the tank valve after the elapsed time. Mass can be corrected in overfilled devices by safely venting some of the working fluid through the attached filling valve.

## 8 Conclusion

This paper describes in detail hardware for the vacuum evacuation, degassing and charging of working fluids for the manufacture of two-phase thermal management and control devices, such as heat pipes, thermosyphons, or vapour chambers. It is designed as a single bench-top station for use in a laboratory environment for the rapid development and prototyping of two-phase heat transfer devices. Two configurations of the system allow for the filling of working fluids in the range of 1–25 mL which are either liquid or gases at standard temperature and pressure conditions. It is intended to be highly customisable and scalable whereby it can be modified to meet the filling volume requirements of the heat transfer test device as the schematics and operation instructions remain the same independent of size. Components such as the pressure transducers, burette, tanks and fittings can be exchanged to adapt to a particular application.

Characterisation of the hardware was performed through the manufacture of multiple thermosyphon two-phase devices for a variety of common working fluids, including water, acetone and ammonia. For both configurations, very good filling volume precision and accuracy was achieved within an average of 0.11 g of the target quantity. This corresponds to average values of 1.7% and 10.5% compared to the target masses for liquids and gases respectively.

**Nomenclature**

**Table:** 

d	Diameter [m]
$H_{S}$	Henry’s Law solubility constant [Pa^−1^]
l	Length [m]
M	Molecular weight [kg/mol]
P	Pressure [Pa]
R	Specific gas constant [J/(kg K)]
T	Temperature [°C]
t	Wall thickness [m]
V	Volume [m^3^]
*Greek*	
ρ	Density [kg/m^3^]
*Subscripts*	
a	Adiabatic
c	Condenser
e	Evaporator
g	Gas phase
i	Index
l	Liquid phase
tot	Total
v	Vapour

## CRediT authorship contribution statement

**Colin Butler:** Writing – original draft, Validation, Software, Project administration, Methodology, Investigation, Funding acquisition, Data curation, Conceptualization. **Emmanuel Caplanne:** Writing – review & editing, Supervision, Project administration, Conceptualization. **Jeff Punch:** Writing – review & editing, Supervision, Project administration, Conceptualization.

## Declaration of competing interest

The authors declare that they have no known competing financial interests or personal relationships that could have appeared to influence the work reported in this paper.

## Figures and Tables

**Fig. 9:**
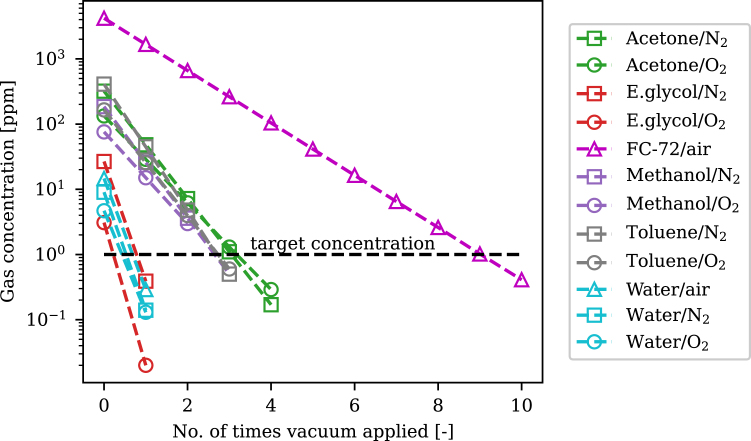
Vacuum degassing of liquids.

**Table 4:** Henry’s Law constants for selected liquids.

Liquid	Gas	T [°C]	$P_{v}$ [mbar]	$H_{S}$ × 10^9^ [Pa^−1^]
Acetone	N_2_	25	307	5.35 [18]
	O_2_			8.29 [19]
Ethylene glycol	N_2_		0.1	0.34 [18]
	O_2_			0.15 [20]
FC-72	Air		309	54 [12]
Methanol	N_2_		170	2.69 [18]
	O_2_			4.10 [19]
Toluene	N_2_		38	5.34 [18]
	O_2_			7.99 [19]
Water	N_2_		32	0.12 [18]
	O_2_			0.23 [19]
	Air	20	23	0.15 [18]

## Appendix Vacuum degassing of liquids

Any non-condensable gas dissolved in the working fluid must be removed before it is filled into a two-phase device. The method for determining the concentration of air in the liquid working fluid is detailed below, and involves repeatedly pulling a vacuum on the vapour/air above the liquid in a technique known as vacuum degassing. The advantage of this technique is that the pressure in the degassing tank is never below the vapour pressure of the liquid [12]. When selecting a vacuum pump for this task, its ultimate pressure therefore needs only to be lower than the vapour pressure of the working fluid, meaning high performance vacuum pumps are not always required to thoroughly degas the liquid.

The dissolved gas concentration $C_{g}$ in the liquid phase is given by Henry’s Law,

(A.1) $$C_{g}=H_{S}P_{g}$$

where $H_{S}$ is Henry’s Law solubility constant as a function of temperature [17] and $P_{g}$ is the partial pressure of gas above the liquid. Values of $H_{S}$ are listed in Table 4. The value of $P_{g}$ can be determined from measuring the total pressure $P_{tot}$ and temperature above the liquid after it has come to equilibrium in a sealed container from the following equation,

(A.2) $$P_{g}=P_{tot}-P_{v}\left(T\right)$$

where $P_{v}$ is the saturated vapour pressure of the fluid at temperature T. If the vapour and gas are removed using the vacuum pump, assuming the liquid volume does not change, and allowing the chamber to equilibrate, the gas dissolved in the liquid must come out of solution to fill the evacuated volume. The total mass of gas in the container is therefore the sum of gas within and above the liquid, given by,

(A.3) $$H_{S}P_{g,i}\rho_{l}V_{l}\frac{M_{g}}{M_{l}}=\frac{P_{g,i+1}}{RT}V_{g}+H_{S}P_{g,i+1}\rho_{l}V_{l}\frac{M_{g}}{M_{l}}$$

where $P_{g,i}$ is the initial partial pressure of the gas above the liquid, $P_{g,i+1}$ is the partial pressure of the gas after vacuum has been applied, $\rho_{l}$ is the density of the liquid, $V_{l}$ and $V_{g}$ are the liquid and gas volumes respectively, $M_{g}$ and $M_{l}$ are the molecular weights of the gas and liquid respectively, and R is the specific gas constant. Rearranging Eq. (A.3) as

(A.4) $$\frac{P_{g,i+1}}{P_{g,i}}=\frac{H_{S}\rho_{l}V_{l}\frac{M_{g}}{M_{l}}}{\frac{V_{g}}{RT}+H_{S}\rho_{l}V_{l}\frac{M_{g}}{M_{l}}}$$

gives the reduction in partial pressure of the gas above the liquid due to the removal of the gas and working fluid vapour above the liquid. From knowing the initial conditions, it is therefore possible to determine the number of times the vacuum needs to be applied to reduce the gas concentration to below a certain concentration.

The results of this method are demonstrated in Fig. 9 for the liquids given in Table 4. To reduce the gas concentrations to less than 1 ppm, assuming the degassing flask is half filled with liquid ($V_{g}=V_{l}$), and for an initial total pressure of 1013 mbar, it can be seen that a vacuum needs to be applied at least one time for water, three times for methanol and toluene, four times for acetone, and ten times for FC-72.

As mentioned early, this analysis suggests that a high performance vacuum pump is not required for liquid degassing. For acetone with a vapour pressure of 307 mbar, a mini diaphragm pump with an ultimate pressure of 160 mbar (estm. €350) could potentially be used. For liquids with lower vapour pressures, such as water or toluene, a single-stage rotary vane pump with an ultimate pressure of 0.3 mbar would be required (estm. €1,700) instead.

However, another important consideration for vacuum pump selection includes the evacuation of the test device before filling. Using for example the longest sample from Table 2, if the vacuum pressure is reduced to 0.3 mbar, the remaining moles of gas can be calculated from the ideal gas law and the sample’s internal volume. Dividing this by the working fluid filling volume (in moles) gives an impurity level of 3 ppm after filling. By using a two-stage rotary vane pump with an ultimate vacuum level of 2 × 10^−3^ mbar (estm. €2,400), this impurity level can be lowered to 0.02 ppm after filling. A trade-off is therefore necessary between the acceptable impurity levels of the working fluid inside the two-phase device and the vacuum pump cost.
